# Verification of the electromagnetic deep-penetration effect in the real world

**DOI:** 10.1038/s41598-021-95080-w

**Published:** 2021-08-05

**Authors:** Paolo Baccarelli, Alessandro Calcaterra, Fabrizio Frezza, Fabio Mangini, Nicholas Ricciardella, Patrizio Simeoni, Nicola Tedeschi

**Affiliations:** 1 Department of Engineering, Roma Tre University, 00146 Rome, Italy; 2 Department of Information Engineering, Electronics and Telecommunications (DIET), Sapienza University of Rome, 00184 Rome, Italy; 3 Elt Elettronica Group, 00131 Rome, Italy; 4 Department of Information Engineering, University of Brescia, 25123 Brescia, Italy; 5 National Transport Authority, Dublin, Ireland

**Keywords:** Engineering, Electrical and electronic engineering

## Abstract

The deep penetration of electromagnetic waves into lossy media can be obtained by properly generating inhomogeneous waves. In this work, for the very first time, we demonstrate the physical implementation and the practical relevance of this phenomenon. A thorough numerical investigation of the deep-penetration effects has been performed by designing and comparing three distinct practical radiators, emitting either homogeneous or inhomogeneous waves. As concerns the latter kind, a typical Menzel microstrip antenna is first used to radiate improper leaky waves. Then, a completely new approach based on an optimized 3-D horn TEM antenna applied to a lossy prism is described, which may find applications even at optical frequencies. The effectiveness of the proposed radiators is measured using different algorithms to consider distinct aspects of the propagation in lossy media. We finally demonstrate that the deep penetration is possible, by extending the ideal and theoretical evidence to practical relevance, and discuss both achievements and limits obtained through numerical simulations on the designed antennas.

## Introduction

Nowadays, electromagnetic fields are used for many applications: to bring information, as happens in communications systems, in radar applications, in electronic measurements, and in the imaging and spectroscopy of human body tissues, or to simply deliver power, as happens in the heating of foods or in several medical treatments such as radiotherapy.

This is due to the high versatility of the electromagnetic fields, that bring with them raw power as long as signals that can trace any discontinuity that the field may find.

Anyway, there are physical constraints that limit the applicability of electromagnetic fields, such as the attenuation offered by media in which they are travelling. Moreover, many of the media we are interested to pass through offer high attenuation.

For example, being able to overcome these limits, propagating deeper and deeper in such media, for the Ground Penetrating Radar (GPR)^1^ would mean being able to electrically scan wider areas, thanks to reflections that occur from deeper objects^2^. For tumor ablation, it would mean being able to treat wider cancerous tissues, due to the power delivered more in depth and less attenuated^3^.

In the literature^4,5^ there are analytical approaches to enhance the propagation in lossy media using plane waves: this phenomenon is simply called “deep-penetration effect”. Herein, we propose and demonstrate a nontrivial way to achieve this fundamental result, by nevertheless considering a real scenario with the use of practical radiating elements, numerically modelled with an electromagnetic commercial software, i.e. CST MW Studio 2019 (www.cst.com).

We will consider three different antennas. The first one is a classical horn antenna^6^ radiating a homogeneous wave. The second one belongs to the family of leaky-wave antennas (LWAs)^7,8^. Eventually, the horn TEM with a lossy prism^9^ (henceforth named HTwLP) will be considered. This structure also produces non-homogeneous waves since an attenuation vector is introduced by the losses of the prism medium. After some considerations on the characteristics of the inhomogeneous waves produced by the LWA and the HTwLP, we will show how these antennas can generate fields that propagate much more than the one produced by the horn, thus making deep penetration achievable with real antennas.

It is well known^10^ that electromagnetic waves propagating in a medium are composed of time and space-variant electric ($\underline {E}$) and magnetic ($\underline {H}$) fields, that in order to satisfy the Helmholtz equation (derived from the Maxwell equations in a homogeneous region with no sources) must assume the form $\underline {A} = { }\underline {A}_{0} {\text{exp}}\left[ { - j\left( {\underline {k} \cdot \underline {r} - \omega t} \right)} \right]$ where $\underline {A}$ and $\underline {A}_{0}$ represent either $\underline {E}$ or $\underline {H}$. The constant vector $\underline {A}_{0}$ contains the polarization characteristics, while the exponential factor determines the propagation features. In the wave equation, we defined with $j$ the imaginary unit number $\left( {j^{2} = - 1} \right)$, with $t$ the time variable, $\omega$ the angular frequency, $\underline {r}$ the position vector, and $\underline {k}$ the wave vector. The latter is a complex vector connected to both the medium properties (in which the wave is propagating) and the frequency. Furthermore, $\underline {k}$ can be thought as a combination of two real vectors, the phase vector $\underline {\beta }$, and the attenuation vector $\underline {\alpha }$ through the expression $\underline {k} = \underline {\beta } - j\underline {\alpha }$. A wave in a lossy medium is said homogeneous, or uniform, when phase and attenuation vectors have the same direction, otherwise is said inhomogeneous^10–12^. In lossless media, the attenuation vector is either zero $\left( {\underline {k} = \underline {\beta } } \right)$, or it is orthogonal to the phase vector, i.e., $\underline {\alpha } \bot \underline {\beta }$. In a lossy medium, the attenuation vector must exist and, in this case, $\widehat{{\underline {\alpha } \underline {\beta } }} = \theta < \pi /2$ rad. In other words, the electromagnetic-wave radiation may or may not have an attenuation in a certain direction if the medium is lossless, while such an attenuation must always exist if the medium is dissipative, as one would expect.

**Figure 1:**
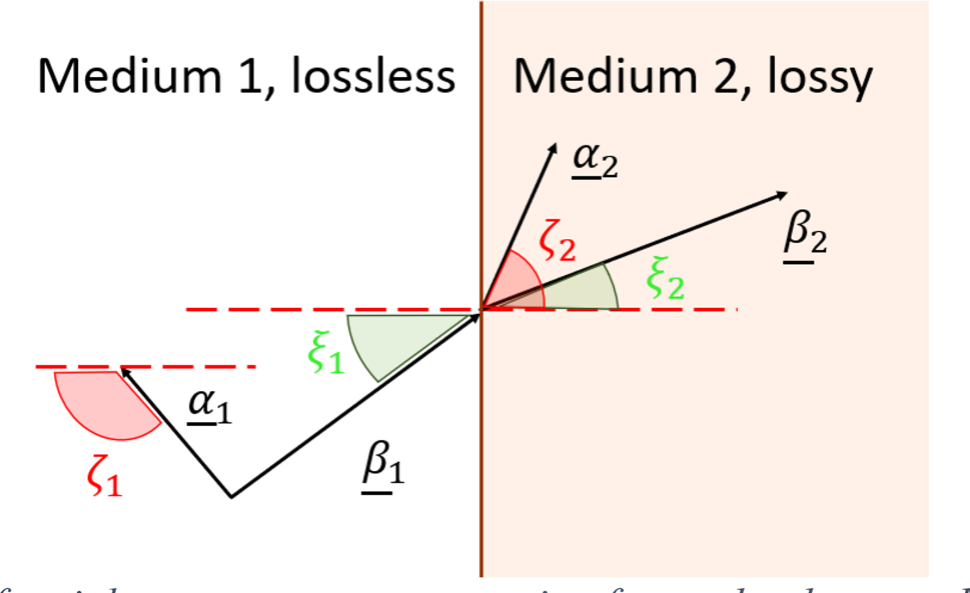
Case of incidence of an inhomogeneous wave coming from a lossless to a lossy medium. The interface between the media is planar, highlighted in red in the picture.

Let us consider a lossless medium, that we indicate here as “Medium 1” and a lossy medium, indicated here as “Medium 2”, separated by an infinite planar interface (see Fig. 1). Let us assume a plane wave (called in the literature “incident wave”) coming from Medium 1 and incident on the interface with Medium 2. Then, a plane wave is transmitted in Medium 2 and reflected in Medium 1; here we are interested in the analysis of the transmitted wave. We indicate with $\underline {k}_{1} = \underline {\beta }_{1} - j\underline {\alpha }_{1}$ and $\underline {k}_{2} = \underline {\beta }_{2} - j\underline {\alpha }_{2}$ the wave vectors of the incident and transmitted waves, respectively, where $\underline {\beta }_{1}$ and $\underline {\alpha }_{1}$ are phase and attenuation vectors for the incident wave and $\underline {\beta }_{2}$ and $\underline {\alpha }_{2}$ are phase and attenuation vectors for the transmitted wave, maintaining the conventions introduced in^4,9,13,14^.

In^5^, extending and completing the results found in^4^, the authors demonstrate that, employing inhomogeneous plane waves, the normal component of the attenuation of the transmitted wave can be completely cancelled, allowing for infinite penetration: the minimal condition which permits deep penetration is the one that guarantees $\zeta_{2c} = \zeta_{2} = {\uppi }/2$ rad, and it is obtained for a critical value ${\xi }_{c}$ of the incidence angle ${\xi }_{1}$ defined either as:

1 $${\xi }_{c}=\frac{1}{2}arcsin\left[\frac{Im\left({k}_{2}^{2}\right)}{{\beta }_{1}{\alpha }_{1}}\right]$$

or as:

2 $${\xi }_{c}=\frac{\pi }{2}-\frac{1}{2}arcsin\left[\frac{Im\left({k}_{2}^{2}\right)}{{\beta }_{1}{\alpha }_{1}}\right]$$

depending on the characteristics of the media involved in the problem. In^11^ (1) and (2), we indicated with $\beta_{1} = \left| {\underline {\beta }_{1} } \right|$ the amplitude of $\underline {\beta }_{1}$, with $\alpha_{1} = \left| {\underline {\alpha }_{1} } \right|$ the amplitude of $\underline {\alpha }_{1}$, and with $k_{1} = \sqrt {\underline{{k_{1} }} \cdot \underline{{k_{1} }} }$; analogously, $\beta_{2}$, $\alpha_{2}$ represent the amplitudes of the vector quantities $\underline {\beta }_{2}$, $\underline {\alpha }_{2}$, respectively, and $k_{2} = \sqrt {\underline{{k_{2} }} \cdot \underline{{k_{2} }} }$.

The value $\xi_{c}$ is found for $\beta_{1} \ge \beta_{c}$ defined as follows:

3 $${\beta }_{c}=\frac{{k}_{1}}{\sqrt{2}}\sqrt{1+\sqrt{1+{\left[\frac{2Im\left({k}_{2}^{2}\right)}{{k}_{1}^{2}}\right]}^{2}}}$$

In correspondence of ${\beta }_{c}$, we will have an ${\alpha }_{c}$ equal to:

4 $${\alpha }_{c}=\frac{{k}_{1}}{\sqrt{2}}\sqrt{-1+\sqrt{1+{\left[\frac{2Im\left({k}_{2}^{2}\right)}{{k}_{1}^{2}}\right]}^{2}}}$$

The study^5^ also demonstrates the feasibility of a larger penetration, i.e., ${\zeta }_{2}\ge {\zeta }_{2c}$ and it shows how the penetration pattern changes employing different permittivity values for both Medium 1 and Medium 2: all media considered in^5^ are non-magnetic, i.e., ${\mu }_{1}={\mu }_{2}={\mu }_{0}$, where ${\mu }_{0}$ represents the vacuum permeability. We will also consider non-magnetic media in the simulations presented in the following section.

Three categories of realistic-inhomogeneous waves are well known at the present days, and they can potentially be investigated for this objective: the surface wave, the lateral wave, and the leaky wave^15,16^. Among those, the only wave which is not bonded to the separation surface between the media is the leaky wave, and therefore it represents the only known mean of achieving deep penetration. Furthermore, in^5^ it is demonstrated that only the leaky wave of the improper type is allowed for deep penetration, while that of the proper type, typically present in metamaterial LWA^17,18^, is shown to be unsuitable.

Another way to generate inhomogeneous waves, that differs significantly from the one cited above, involves the presence of losses in the medium. This approach was introduced in^9^ and foresees a primary source (for instance, a horn antenna) radiating a homogeneous wave toward a lossy prism. Once the primary wave has impinged on the prism, the transmitted wave, that travels into the prism, must possess an attenuation vector^10,12^ due to the losses in the material, and once it reaches the end of the prism, if the surface is not parallel to the first one, the tangential component of this attenuation vector will be conserved^19,20^, generating another transmitted inhomogeneous wave, this time in the lossless space.

With these two different approaches, through an LWA generating an improper leaky wave^5^ and through a horn TEM with a lossy prism (HTwLP) to generate an inhomogeneous wave, we will verify the deep-penetration effect.

LWAs generate electromagnetic radiation through the continuous leakage of power along the aperture^7,8^, producing a wave that can be, in the near-field region, approximated by an inhomogeneous plane wave. In particular, uniform, non-periodic LWAs emit improper leaky waves, which are best suited for our goal. LWAs have already been proposed in the literature to improve  electromagnetic penetration in hyperthermia treatment^21^ and ultra-high field magnetic resonance imaging^3^ leading to an improvement of the near-field effects. Here, we exploit and widen the theoretical results provided in^5^ to prove applicability and limits of the deep-penetration equations in realistic scenarios, by using electromagnetic full-wave numerical simulations.

The antenna chosen for the first numerical experiment is the well-known Menzel Antenna^22–25^, a simple uniform microstrip antenna.

**Figure 2:**
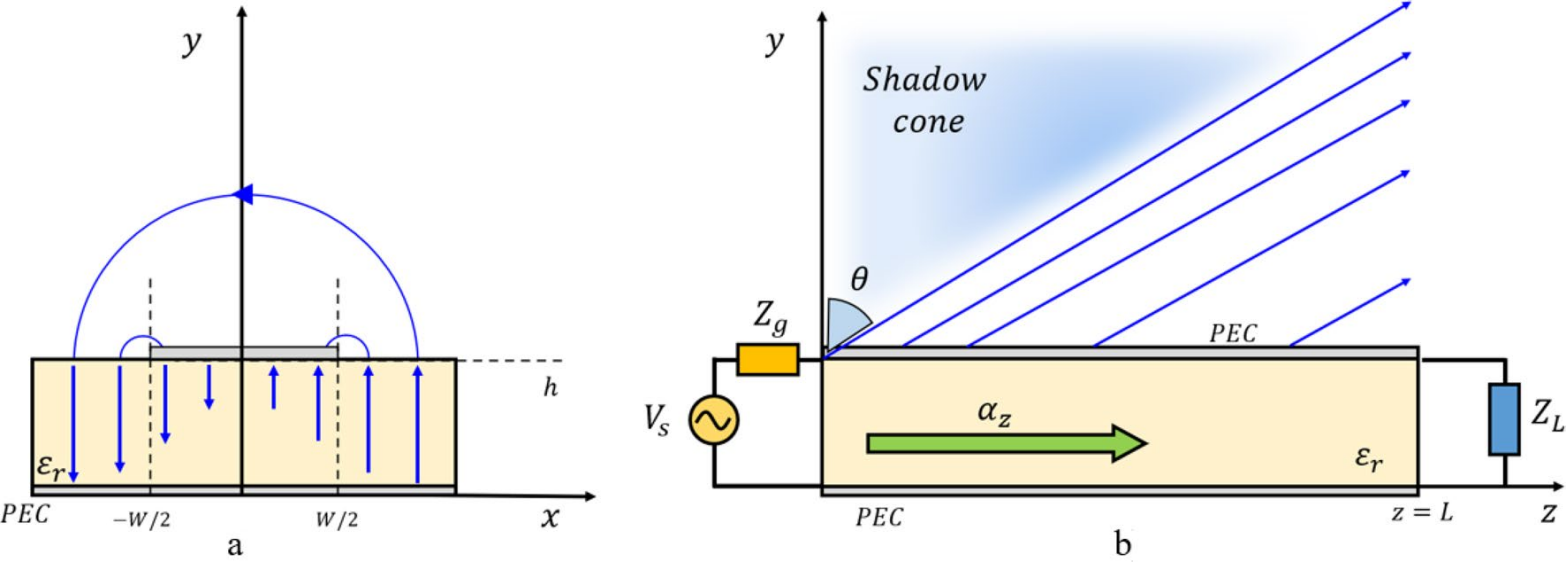
(**a**) Menzel Antenna design principle, cross-section view, the top metal layer (microstrip) has a width of W, and a thin dielectric substrate of height h, whose relative permittivity is ${\upvarepsilon }_{\mathrm{r}}>1$, separates it from the ground plane. (**b)** Longitudinal view: the antenna has length L, and it is fed at z = 0 and closed to a matched load at z = L. In the figure, the radiation due to the first higher-order (EH_1_) mode is illustrated.

The microstrip antenna can radiate through its high-order modes^26^, e.g., the EH_1_ mode, see Fig. 2, acting as an LWA, since its fundamental mode is mainly confined in the substrate. The Menzel antenna represents a good candidate for the study of the deep-penetration effect, not only for its simplicity but mainly because it can generate leaky waves with the desired phase vector $\underline{\beta}_{1}$ and with enough large amplitudes of the attenuation vector $\underline{\alpha}_{1}$, as required in Eqs. (3) and (4) by increasing the conductivity of the lossy medium^5^.

The HTwLP was proposed in^9^ and further investigated in^19,20^. The analysis performed in^19^ shows that $\underline{\alpha}_{1}$ of the generated inhomogeneous wave can be improved by increasing the $\sigma$ of the prism, this comes to a cost since some of the power emitted by the antenna gets dissipated. Equations (5) and (6) show the relationship between the phase and attenuation vectors in the air of the generated inhomogeneous wave depending on the characteristics of the lossy prism and on the ${\xi }_{air}$, the angle formed by the phase vector and the normal to the second interface between the prism and the air.

Note that the only hypothesis to achieve those relations is ${\chi }_{prism}$ $=\pi /2$, which leads to

5 $$\frac{{\beta }_{air}}{{k}_{0}}=\frac{1}{\sqrt{2}}\sqrt{1+\sqrt{1+{\left(\frac{2{\sigma }_{prism}}{\upomega {\upvarepsilon }_{0} \mathrm{sin}\left(2{\xi }_{air}\right)}\right)}^{2}}}$$

6 $$\frac{{\alpha }_{air}}{{k}_{0}}=\frac{1}{\sqrt{2}}\sqrt{-1+\sqrt{1+{\left(\frac{2{\sigma }_{prism}}{\upomega {\upvarepsilon }_{0} \mathrm{sin}\left(2{\xi }_{air}\right)}\right)}^{2}}}$$

In^20^ was shown how the HTwLP can be a good candidate for the deep penetration, even if a simple two-dimensional model was considered. In this paper, a realistic structure will be investigated.

## Results

### Description of the setup

**Figure 3:**
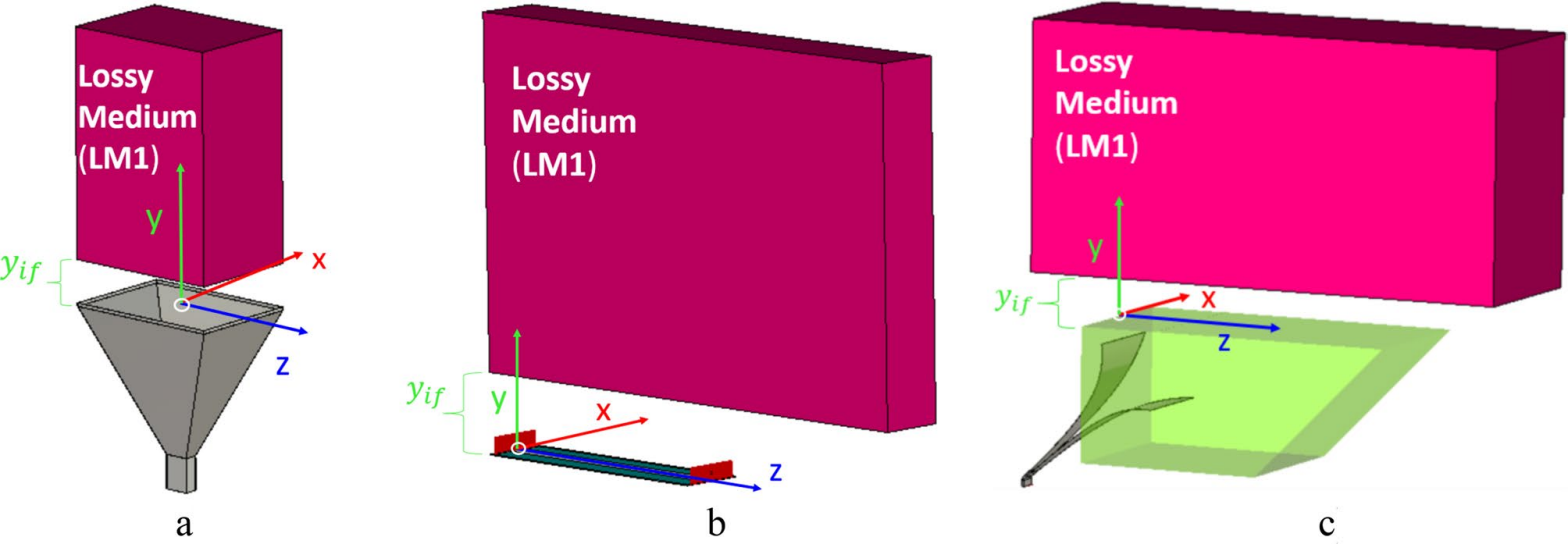
Design of (**a**) horn antenna, (**b**) Menzel antenna, and (**c**) horn TEM with lossy prism in front of the lossy medium on CST.

We compare the field produced by the Menzel antenna and the HTwLP with the one produced by a customary pyramidal horn antenna^6,27^ at the frequency $f=12$ GHz. All the antennas radiate into a vacuum: in close proximity, parallel to the antenna aperture, is placed a lossy parallelepiped invested by this radiation. The apertures of the antennas are positioned on the $y=0$ plane, and the interface with the lossy medium is at $y={y}_{if}$: the value experimentally chosen for ${y}_{if}$ is $1.5\lambda =37.5$ mm, being $\lambda = c{/}f$ the vacuum wavelength, see Fig. 3. Considering the LWA first, when the source is finite, and therefore realistic, the electromagnetic field radiated by the LWA decays exponentially along the longitudinal antenna aperture from its maximum, at the feeding point, here located on the origin of the axes $(0, 0, 0)$. In this scenario, we need to find a region in which the improper-wave behavior exists at the interface with the lossy medium: in other terms, the interface with the lossy medium needs to be outside the shadow cone in which the radiation decays (see Fig. 2).

We designed the Menzel antenna to radiate a leaky wave with a radiation angle $\theta =\uppi /4$ rad: this value of the angle $\theta$ minimizes the amplitudes of both ${\beta }_{1}={\beta }_{1c}$ and ${\alpha }_{1}={\alpha }_{1c}$ that give rise to deep penetration and allows for a practical disposition of the antenna and lossy medium.

The choice taken simplifies the antenna design because an effective non-periodic LWA becomes more challenging as ${\alpha }_{1}$ increases: in particular, the Menzel antenna tends to become shorter and its field tends to decay earlier, thus limiting the region where the inhomogeneous plane wave is present. The objective was to reproduce deep-penetration effect, i.e. ${\zeta }_{2}=\uppi /2$, on a lossy medium, LM1, characterized by parameters ${\varepsilon }_{2}={\varepsilon }_{0}$, ${\mu }_{2}={\mu }_{0}$, i.e. the relative permittivity ${\varepsilon }_{r}$ and the relative permeability ${\mu }_{r}$ are both equal to one, and having conductivity ${\sigma }_{2}=0.05$ S/m.

Preliminary results of the analysis of the Menzel antenna in terms of deep penetration were shown in^28^, in which the agreement between the antenna and theoretical model^5^ was illustrated.

The HTwLP was designed to achieve $\beta ={\beta }_{1c}$ and $\alpha ={\alpha }_{1c}$. Once the ${\sigma }_{prism}$ was chosen according to the desired $\alpha$, the structure was optimized. In this analysis, the upper wedge of the prism $\chi$, according to^19^, was chosen equal to $\pi /2$ rad to maximize the conservation of $\alpha$. The prism was chosen with ${\varepsilon }_{r}={\mu }_{r}=1$ to limit reflections at the prism interfaces, simplifying the numerical analysis. Also, the HTwLP was designed to radiate with an angle of $\pi /4$ rad, as the Menzel antenna. Eventually, the horn antenna chosen presents broadside radiation. All antennas were simulated using the F.I.T. (Finite Integration Method)^29,30^ module implemented in the CST Microwave Studio Software licensed to the DIET Department of “La Sapienza” University of Rome.

### Review of the antenna characteristics in terms of deep-penetration effect

In this section, we will analyze and comment on the behavior of an improper leaky wave impinging at the interface between the air and the lossy medium LM1 and of the relevant transmitted field propagating within the latter. In the following, most of the results will be shown for the LWA, but they can be naturally extended to the HTwLP. In the numerical simulations, we evaluated the electric field on the symmetry plane $x=0$ of all the structures along the longitudinal direction and we exported the amplitudes of the electric-field components for further processing.

As concerns the pyramidal horn antenna, we can evaluate the electric-field trend in the lossy medium considering its normalized value along the *y*-axis (i.e., the antenna symmetry axis), where the electric-field amplitude produced by the antenna is maximum. The field was normalized by its value on the same axis ($x=0$ and $z=0$) at $y={y}_{if}$, i.e. at the interface between air and the LM1.

7 $$\overline{E}\left(y\right)=\frac{\left|\underline{E}\left(0,y,0\right)\right|}{\left|\underline{E}\left(0,{y}_{if},0\right)\right|}$$

It is more difficult to evaluate the penetration on a mono-dimensional curve when the other two antennas are considered because of the $\uppi /4$ incidence angle.

**Figure 4:**
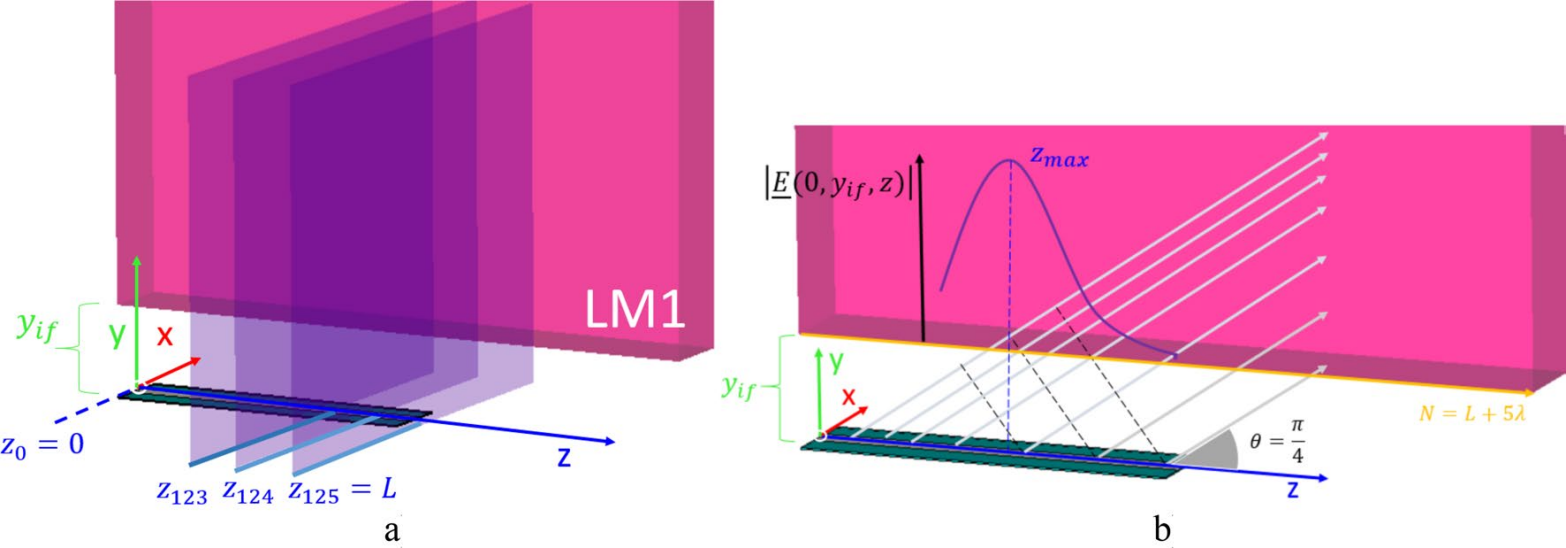
Menzel antenna with (**a**) the transverse planes cutting the antenna orthogonally and (**b**) the radiated field.

For instance, let us start with the LWA case. As shown in Fig. 4, we take $N$ samples on the longitudinal direction of the antenna aperture, i.e. along the $z$-axis, at a sample rate of $1$ mm equal to $\lambda /25$, so that ${z}_{k}$ represents a plane passing by the ${k}_{th}$ point on the longitudinal direction, distant $k$ millimeters from the plane $z=0$ (where the field source is located) and parallel to such a plane. Possible values for ${z}_{k}$ are $0,\dots ,L,L+1,\dots ,N-1,N$ where $z=0$ is the plane passing through the feeder and $z=L$ corresponds to the plane passing through the termination, where we place an appropriate matched load to absorb the residual radiation, thus preventing reflections from the termination. As a consequence of the radiation angle of $\uppi /4$, $N$ had to be chosen reasonably larger than $L$, and in particular $N=L+5\lambda$, to ensure that the radiation fully impinged on the LM1.

In^28^, we evaluated a dimensionless quantity obtained by normalizing the amplitude of the electric field $E\left(0,y,{z}_{k}\right)$ ($E\left(0,y,{z}_{k}\right)=\left|E\left(0,y,{z}_{k}\right)\right|$) on the longitudinal symmetry plane to the amplitude of the electric field evaluated at $\left(0,{y}_{if},{z}_{k}\right),$ obtaining a family of mono-dimensional curves, one for each value of ${z}_{k}$ considered:

8 $${\left.\overline{E}\left(y\right)\right|}_{{z}_{k}}=\frac{\left|\underline{E}\left(0,y,{z}_{k}\right)\right|}{\left|\underline{E}\left(0,{y}_{if},{z}_{k}\right)\right|}$$

**Figure 5:**
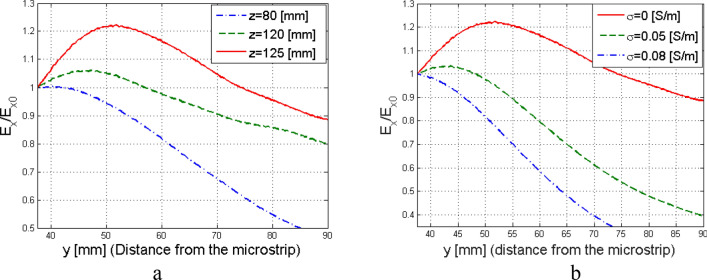
(**a**) Amplitude of the electric field $\overline{\mathrm{E}}(\mathrm{y})$ produced by an LWA in a vacuum and sampled at a point ${\mathrm{p}}_{0}=\mathrm{p}(0,{\mathrm{y}}_{\mathrm{j}},{\mathrm{z}}_{\mathrm{k}})$ normalised to the value of the electric-field amplitude calculated at ${\mathrm{p}}_{\mathrm{if}}=\mathrm{p}\left(0,{\mathrm{y}}_{\mathrm{if}},{\mathrm{z}}_{\mathrm{k}}\right)$; being ${\mathrm{z}}_{\mathrm{k}}>0$ and ${\mathrm{y}}_{\mathrm{j}}>{\mathrm{y}}_{\mathrm{if}}=1.5\uplambda$. (**b**) Amplitude of the electric field E for $\mathrm{z}=5\uplambda$ in three different media with growing conductivity. The LWA was designed such that the attenuation vector would have been parallel to the separation surface for $\upsigma =0.05$ S/m, $\upmu ={\upmu }_{0}$, and $\upvarepsilon ={\upvarepsilon }_{0}$.

Curves describing ${\left.\overline{E}\left(y\right)\right|}_{{z}_{k}}$, for few particular ${z=z}_{k}$ values are illustrated in Fig. 5a: it is possible to verify that the generated electromagnetic wave starts presenting improper behavior for ${z}_{k}\ge 80$ mm.

By reducing the distance ${y}_{if}$, the leaky-wave behavior would have been experienced for ${z}_{k}<80$ mm, but, at the same time, having the medium too close to the antenna could bring it in the reactive region, where the lossy medium becomes part of the antenna itself, changing completely the nature of the electromagnetic radiation: ${y}_{if}=1.5\lambda$ was demonstrated to be a good compromise between the two, opposite, requirements (see “Methods”).

The antenna was designed to achieve $\zeta_{2} = \pi {/2}$ on LM1: from^5^ it is expected that the inhomogeneous wave amplitude value remains constant propagating into LM1 and that, maintaining constant permittivity ${\epsilon }_{2}$ and permeability ${\mu }_{2}$, the inhomogeneous wave attenuates (${\zeta }_{2}<\uppi /2$) when $\sigma_{2}^{\prime \prime } > 0.05$ S/m and its amplitude increases (${\zeta }_{2}>\pi /2$) if $\sigma_{2}^{\prime \prime } < 0.05$ S/m. The behavior demonstrated for plane waves was confirmed here through the numerical simulations: the amplitude of the normalized E-field indicator as a function of the height *y* is shown for ${z}_{k}=125$ mm $=5\lambda$, confirming the preliminary results illustrated in^28^, reported here for completeness in Fig. 5b.

**Figure 6:**
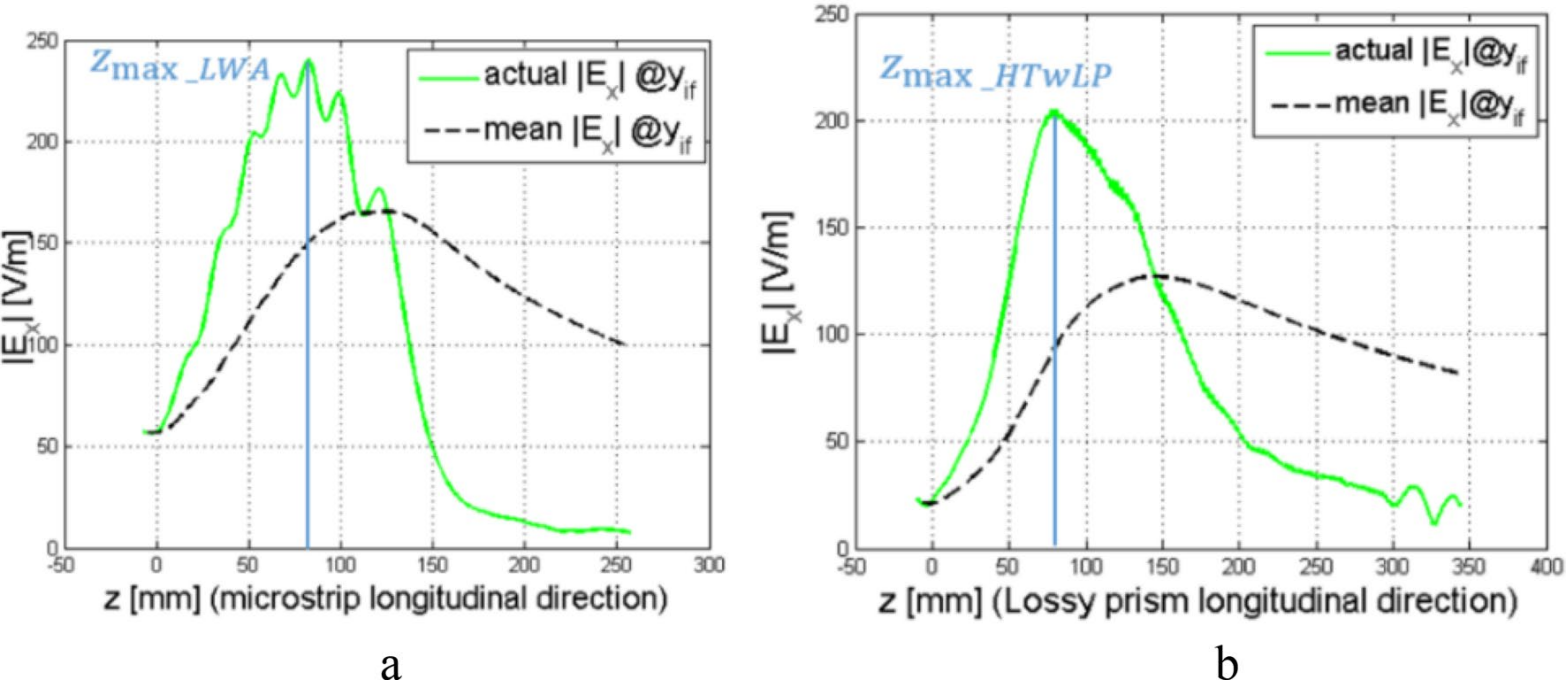
Amplitude of the electric field at the separation interface placed at the distance $1.5\uplambda$ from the antenna aperture, the actual field value (${\mathrm{E}}_{\mathrm{x}}$) is shown in green, while the averaged value is shown in black: (**a**) for the LWA and (**b**) for the HTwLP. In blue, the ${\mathrm{z}}_{\mathrm{max}}$ at which the maximum value of the actual field is indicated in both figures.

This representation gives a good intuitive description of the electric field produced by the LWA, but such a description, according to Eq. (8) becomes critical when ${z}_{k}$ approaches values larger than $L$, because the amplitude of the electric field at ${y}_{if}$ becomes negligible and therefore ${\left.\overline{E}\left(y\right)\right|}_{{z}_{k}}$ tends to diverge: this is apparent in Fig. 6a (green curve), where the amplitude of the electric field at the interface with LM1 is illustrated.

Here, we see a quickly growing field, which remains almost constant first, and then decreases with a large slope: this behavior is a direct effect of the finiteness of the antenna. The contributions to the field due to the electric field leaked in the initial sections of the antenna aperture start summing up to a maximum, reached at ${z}_{max}$. Then, those contributions become exponentially weaker with the distance from the source, since the power is radiated at a constant rate along the aperture until the remained field is totally absorbed by the load placed at $z=L$. This is also visible in Fig. 4, where the trend of the field at the interface, i.e. for ${y=y}_{if}$, is qualitatively shown in a solid purple line.

In Fig. 6a, ${z}_{max}$ also represents the border of the shadow cone. For $y>{y}_{if}$ and $z>{z}_{max}$, the radiated field behaves as an improper leaky wave.

### Comparison between horn antenna, LWA, and HTwLP in terms of electric-field penetration

To numerically validate the deep-penetration effect, we considered a field amplitude normalization able to take into account the different angles of power propagation in the three scenarios (horn, Menzel, and HTwLP antenna):

9 $$\left. {\overline{\overline{E}}\left( y \right)} \right|_{{z_{k} }} = \frac{{\left| {\underline {E} \left( {0,y,z_{k} } \right)} \right|}}{{\mathop \sum \nolimits_{j = 0}^{j = k} \left| {\underline {E} \left( {0,y_{if} ,z_{j} } \right)} \right|/\left( {k + 1} \right)}}$$

At the denominator, all the amplitudes of the electric field for ${z}_{j}<{z}_{k}$ are averaged because they are assumed to contribute to the electric-field amplitude observed at the point $P\left(0,y,{z}_{k}\right)$, where ${y>y}_{if}$. The curve describing the average electric-field amplitude at the interface between lossy medium and vacuum, as a function of ${z}_{k}$, which is the denominator of Eq. (9), is displayed in black in Fig. 6a for the Menzel antenna and in Fig. 6b for the HTwLP. Note that for the pyramidal horn, the result is trivial: due to the symmetry of the structure and the fact that the antenna is radiating at the boresight, the ${z}_{max}$ for the horn (that is the *z* value at which we have the maximum of the electric field when $x=0, y={y}_{if}$) occurs at $z=0$ mm.

**Figure 7:**
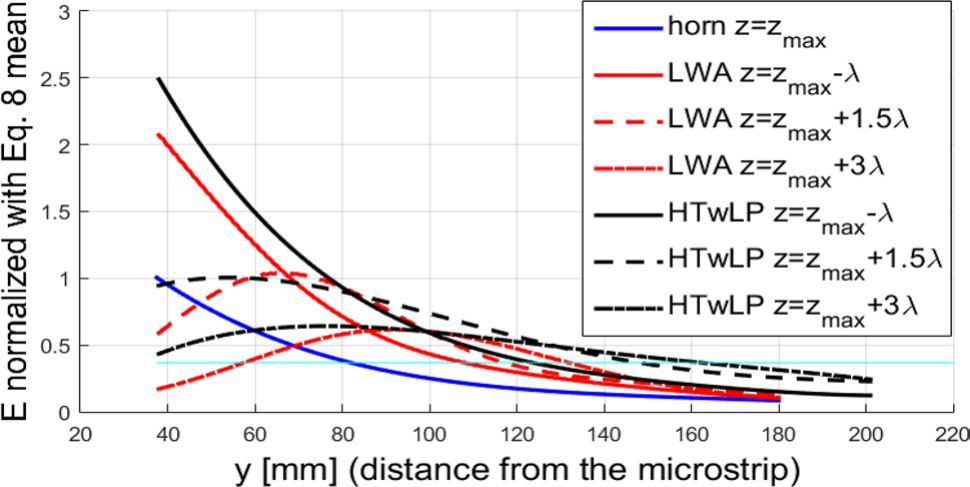
Comparison between horn antenna, LWA, and HTwLP penetration according to Eq. (9). The horizontal line represents the value $\frac{1}{\mathrm{e}}$. For each antenna, ${\mathrm{z}}_{\mathrm{max}}$ is the value of $\mathrm{z}$ at which the maximum absolute value of the field when $\mathrm{y}={\mathrm{y}}_{\mathrm{if}}$ occurs. Note that for the horn ${\mathrm{z}}_{\mathrm{max}}$, due to the symmetry, is simply placed at $\mathrm{z}=0$.

In Fig. 7, $\left. {\overline{\overline{E}}\left( y \right)} \right|_{{z_{k} }}$ curves for few ${z}_{k}$ values compared with the amplitude of the electric field produced by the horn antenna, normalized as described in Eq. (9) (blue line), are shown. The horizontal cyan line represents the penetration factor $\delta = 1{/}e$.

It is visible how these averaged curves depend on *z*. For this reason, to simplify the comparison between the three antennas, we decided to refer to the curves by the distance from the ${z}_{max}$, that is the value of *z* at which the maximum value of the |$E\left(0, {y}_{if}, z\right)|$ occurs for any antenna (see Fig. 6). It is clearly visible how impinging with an inhomogeneous wave allows us to reach a much deeper relative penetration, with respect to the case of the standard horn antenna (i.e., homogeneous case). Also, the HTwLP shows a similar behavior with respect to the LWA: the trend is the same, but the obtained penetration, with respect to the field at the interface, is deeper, thus numerically confirming the effectiveness of the selected inhomogeneous waves in obtaining deep penetration.

An alternative choice for comparing the penetration achieved in the chosen lossy medium through the electric field produced by the antennas, is to normalize the amplitude of the electric field at any point $P(0,y,z)$ inside the medium by the maximum amplitude of the electric field at the interface between lossy and lossless medium:

10 $$\left. {\overline{\overline{E}}\left( y \right)} \right|_{{z_{k} }} = \frac{{\left| {\underline {E} \left( {0,y,z_{k} } \right)} \right|}}{{max_{j} \left| {\underline {E} \left( {0,y_{if} ,z_{j} } \right)} \right|}}$$

**Figure 8:**
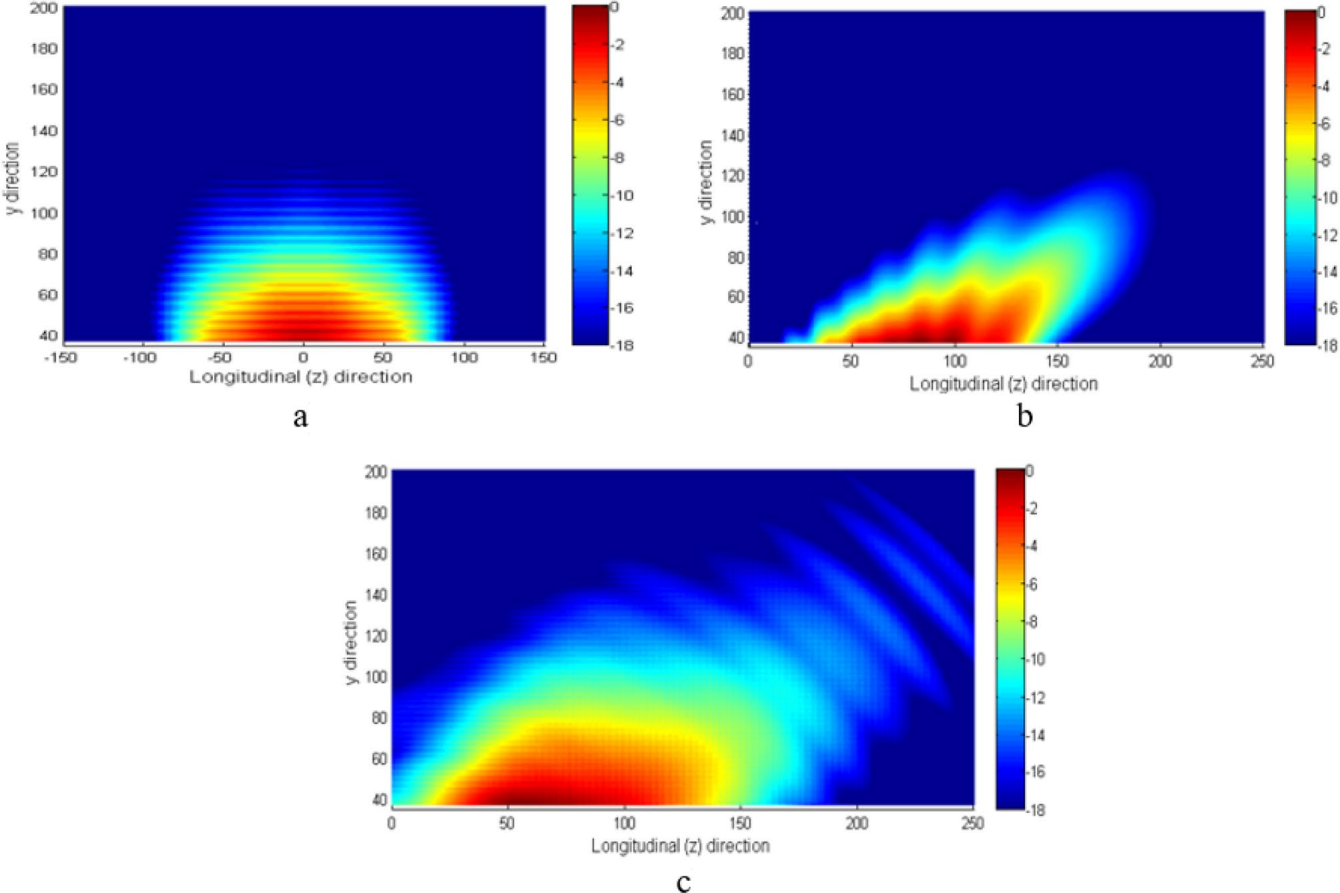
Electric field in the lossy medium produced by the (**a**) horn, (**b**) LWA and (**c**) HTwLP. The field was computed in dB from the highest amplitude of the field at the interface. The lossy medium is positioned at y = 37.5 mm. Dimensions are in mm.

The results for all the antennas are shown in Fig. 8.

The comparison between the fields in the lossy medium shows how antennas providing non-homogeneous waves allow deeper penetration than the pyramidal-horn antenna, this is verified for larger values of ${z}_{k}$, as expected.

The penetration evaluated as described above gives us certainly an encouraging result, and this is logically the most suitable way to evaluate the penetration in many applications, in which the maximum power at the interface is given as a requirement. All the formulas employed up to now describe the trend of the electric field as a function of the observation point, but it is also important to provide a comparison between the average fields obtained inside the lossy medium: this kind of estimate gives us a broader indication on the penetration produced by the structures as a global effect.

It is easy to compute an electric-field average for the pyramidal-horn antenna, i.e. it is sufficient to compute the average of all samples in a direction parallel to the antenna aperture ($z\in \left[0;140\right]$ mm) for every value of ${y}_{j}$ on the $y$ direction, with ${y}_{j}>{y}_{if}$, i.e.:

11 $$\overline{E}\left({y}_{j}\right)=\frac{{\sum }_{k=0}^{k=N-1}\left|\underline{E}\left(0,{y}_{j},{z}_{k}\right)\right|}{N}, \forall {y}_{j}>{y}_{if}$$

having considered $N$ samples $\left\{{z}_{0},{z}_{1},\dots ,{z}_{N-1}\right\}$ on the $z$-axis parallel to the antenna aperture. However, we note that for the other two antennas the evaluation of this average field is not so immediate, because the field is propagating at an angle of $\pi /4$ rad. Therefore, was developed an algorithm which can be applied to any radiating angle to select the appropriate sample distribution. Let us describe the produced algorithm, looking at the LWA first. We preliminarily verified the field at the termination of the LWA aperture, obtaining:

12 $$\frac{\left|\underline{E}\left(\mathrm{0,0},L\right)\right|}{\left|\underline{E}\left(\mathrm{0,0},0\right)\right|}\cong 0.24$$

Equation (12) shows us that about 24% of the electric field is absorbed by the matched load at the LWA termination, corresponding to an absorbed power $\frac{{P}_{L}}{{P}_{0}}$ of about 6%.

At this point, ${N}_{LWA}$ samples with a sampling rate of $1$ mm are selected between $(\mathrm{0,0},0)$ and $\left(\mathrm{0,0},125\right)$ on the overall radiating aperture. For the pyramidal horn antenna, ${N}_{HORN}$ samples, at a sampling rate of $1$ mm, are chosen on the $z$-axis on the aperture in a symmetrical position from the center of the pyramidal-horn antenna aperture (posed at $z=0$). Those samples were chosen such that the minimal electric field was inside the $24\mathrm{\%}$ of the maximum electric field obtained at $z=0$, i.e. (${E}_{MAX}^{HORN}=\left|{\underline{E}}_{HORN}\left(\mathrm{0,0},0\right)\right|$) analogously to the field radiated by the LWA, in which the field at the termination is 0.24 times the one at the excitation port (see Eq. 12). The same was done for ${N}_{HTwLP}$: samples were chosen at a sampling rate of $1$ mm inside the $24\mathrm{\%}$ of the maximum electric field.

Hence, for every ${y}_{i}$, being ${y}_{i}>{y}_{if}$, the electric-field amplitude was then mediated by taking $N$ samples in the $z$ direction, where $N={N}_{LWA}$ for the LWA microstrip antenna, $N={N}_{HTwLP}$ for the horn-TEM with the lossy prism, and $N={N}_{HORN}$ for the pyramidal horn antenna. For every ${y}_{i}\ge {y}_{if}$, the employed algorithm searches the maximum amplitude of the electric field on the $z$ direction, i.e. it looks for a value ${z}_{M}$ such that:

13 $${|E}_{yi}\left(0,{y}_{i},{z}_{M}\right)|\ge {|E}_{yi}\left(0,{y}_{i},{z}_{i}\right)|\forall {z}_{i}\in \left[0;N\right]$$

After ${z}_{M}$ is found, we look for N samples with steps equal to $1$ mm around ${z}_{M}$. For LWA it means taking the ${N}_{LWA}=125$ samples around the maximum where the absolute value of the electric field is bigger. For the pyramidal horn and the HTwLP it means taking the biggest values of the absolute value of the electric field around ${z}_{M}$ as far as the condition $\left|E\left(0, {y}^{*}, z\right)\right|\ge 0.24\left|E\left(0, {y}^{*}, {z}_{M}\right)\right|$ is verified (${y}^{*}$ stands for the particular value of *y* we are considering).

The implemented algorithm, clearly, does not assume any field symmetry around the maximum value of the amplitude of the electric field resulting in a more accurate estimation than the one that could be achieved just assuming the field symmetric around ${z}_{M}$.

**Figure 9:**
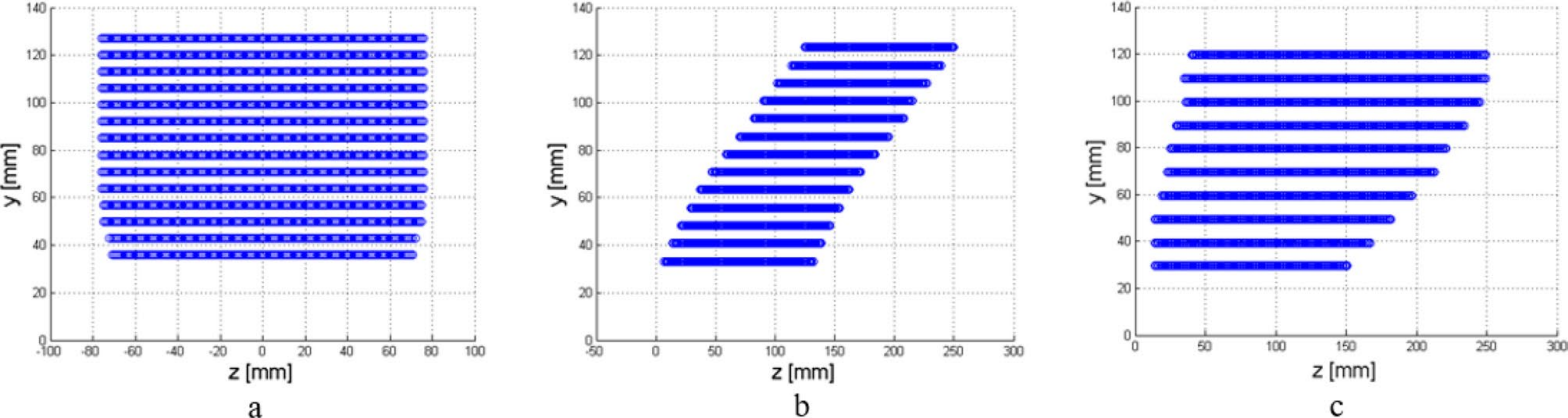
Chosen samples for (**a**) horn, (**b**) LWA microstrip, and (**c**) HTwLP to compute the average penetration for antenna structures. Samples were chosen according to the radiation diagram in a vacuum.

Also, for the pyramidal horn, one could think to assume ${z}_{M}$ to be always 0, which is on the symmetry axis of the structure where the horn is prevalently radiating, or, for the LWA and the HTwLP, that ${z}_{M}$ moves with an angle of $\uppi /4$ with the normal to the separation surface. We rejected these approximations to achieve results free from hypotheses on the field shape. In particular, the samples on the $z$-axis are not in general aligned for ${y}_{i}\ne {y}_{j}, \mathrm{i}.\mathrm{e}., {z}_{{n}^{\left(i\right)}}\ne {z}_{{n}^{(j)}}$.The process is repeated for every ${y}_{i+1}={y}_{i}+1$ mm in the simulation domain. The described algorithm was implemented, and the samples chosen for the three antennas are shown in Fig. 9. The computed averaged value is then normalized by the relevant sum calculated at the first step, i.e. at the separation surface ${y}_{if}$:

14 $$\overline{E }\left({y}_{i}\right)= \frac{{\sum }_{k={n}^{(i)}}^{k=N+{n}^{(i)}}\left|\underline{E}\left(0,{y}_{i},{z}_{k}\right)\right|}{{\sum }_{j=m}^{j=m+N}\left|\underline{E}\left(0,{y}_{if},{z}_{j}\right)\right|} \forall {y}_{i}\ge {y}_{if}$$

**Figure 10:**
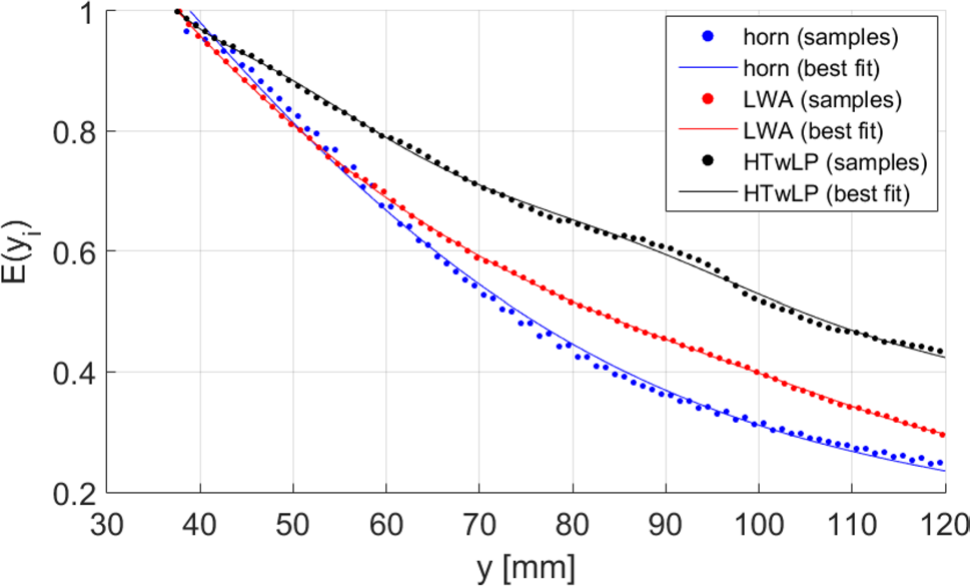
Comparison between horn, LWA, and HTwLP penetrations averaged according to Eq. (14) in a lossy medium for which $\upsigma =0.05\frac{\mathrm{S}}{\mathrm{m}}, {\upmu }_{\mathrm{r}}=1\mathrm{ and }{\upvarepsilon }_{\mathrm{r}}=1.$

Differently from the case seen in Eq. (9) where a family of curves was found, a single curve is obtained. The normalized fields calculated are shown in Fig. 10, where samples are fitted by using a procedure based on the ordinary least square algorithm.

This last comparison gives a very clear indication in terms of the effective electric-field penetration achieved by employing these three different antennas. Again, coherently with the results shown in the previous diagrams, Fig. 10 illustrates that the penetration obtained by means of the horn antenna is higher than that from the LWA and the HTwLP, for low $y$ values. Then, for greater $y$ values, the effect of the leaky-wave improper behavior starts appearing, reducing the slope of the attenuation curve for the LWA, which finally results constantly above the one described by the horn radiation. The field radiated by the HTwLP shows quite a linear slow decay that propagates deeper with respect to both the horn and the LWA inside the lossy medium. These results on the penetrating fields within the lossy medium, averaged on an appropriate number of samples, give a clear and solid indication of the potentiality of the selected (improper) inhomogeneous waves, properly generated by the proposed LWA and HTwLP, in obtaining deep penetration, greatly outperforming homogeneous waves emitted by standard horn antennas.

## Discussion

For the very first time, a possible implementation of the deep-penetration effect is presented by designing two radiators: a low-cost and low-profile Menzel antenna and a 3-D horn TEM antenna applied to a lossy prism.

Through careful design and engineering of the proposed radiators, it is possible to obtain a penetration deeper than that performed by more traditional antennas, such as a standard horn antenna. We compared the fields radiated from the selected structures by using different effective and fair approaches: all these methods confirmed the fundamental finding that impinging with an improper inhomogeneous wave results in the generation of a transmitted wave that is less attenuated with respect to that of a homogeneous one. This work, although verifies the deep-penetration effect for a specific value of the frequency and of the conductivity, being the involved structures typically narrow band, opens avenues to the practical achievement of the deep propagation of electromagnetic waves in lossy media. It paves the way for promising applications in imaging and spectroscopy as well as in radar systems and medical treatments.

Even though the potential for deep penetration is fully demonstrated, the simulations performed also highlighted that reaching large penetrations is subject to some challenges, which might call on more theoretical and experimental efforts in the near future.

In particular, as concerns uniform LWAs, when the conductivity of the lossy medium is increased, then the required values for ${\beta }_{c}$ and ${\alpha }_{c}$ will be greater, as can be seen in Eqs. (3) and (4). Consequently, the antenna longitudinal dimension has to decrease (see Eq. 6) and the propagated field starts decaying earlier. The numerical experiment performed in this paper employed an antenna length of $5\lambda$, for larger values of the conductivity an antenna even shorter would be required, thus reducing the area in which the propagated field behaves as an inhomogeneous wave, and consequently reducing the penetration. The possibility of implementing 2-D LWAs or linear arrays of LWAs^7,8,31^ could be investigated in future studies to emit inhomogeneous waves over a wider area and illuminate bigger volumes of lossy medium.

Furthermore, the HTwLP to penetrate in a more conductive medium needs a more lossy prism: setting the prism parameters as we did, i.e. $\chi =\pi /2$, ${\varepsilon }_{prism}=1, {\mu }_{prism}=1$, it can be shown that the ${\sigma }_{prism}$ must be the same as the medium, i.e., ${\sigma }_{LM1}$. This can be easily obtained by explicating Eq. (6) for ${\sigma }_{prism}$, using the angle of incidence $\xi =\pi /2$ rad and the attenuation ${\alpha }_{c}$ described in Eq. (4). The drawback here is a reduced efficiency, due to higher prism losses. Designing a longer or bigger feeder horn TEM, thus increasing the directivity^8,32^, this effect can be compensated, since the field is more focused: the loss-efficiency remains the same, but the beam efficiency is increased. Otherwise, to keep the same dimensions, it is possible to reduce the path that the wave has to go through into the lossy prism, by reducing $\chi$ and then the component of $\underset{\_}{\alpha }$ that is conserved.

**Figure 11:**
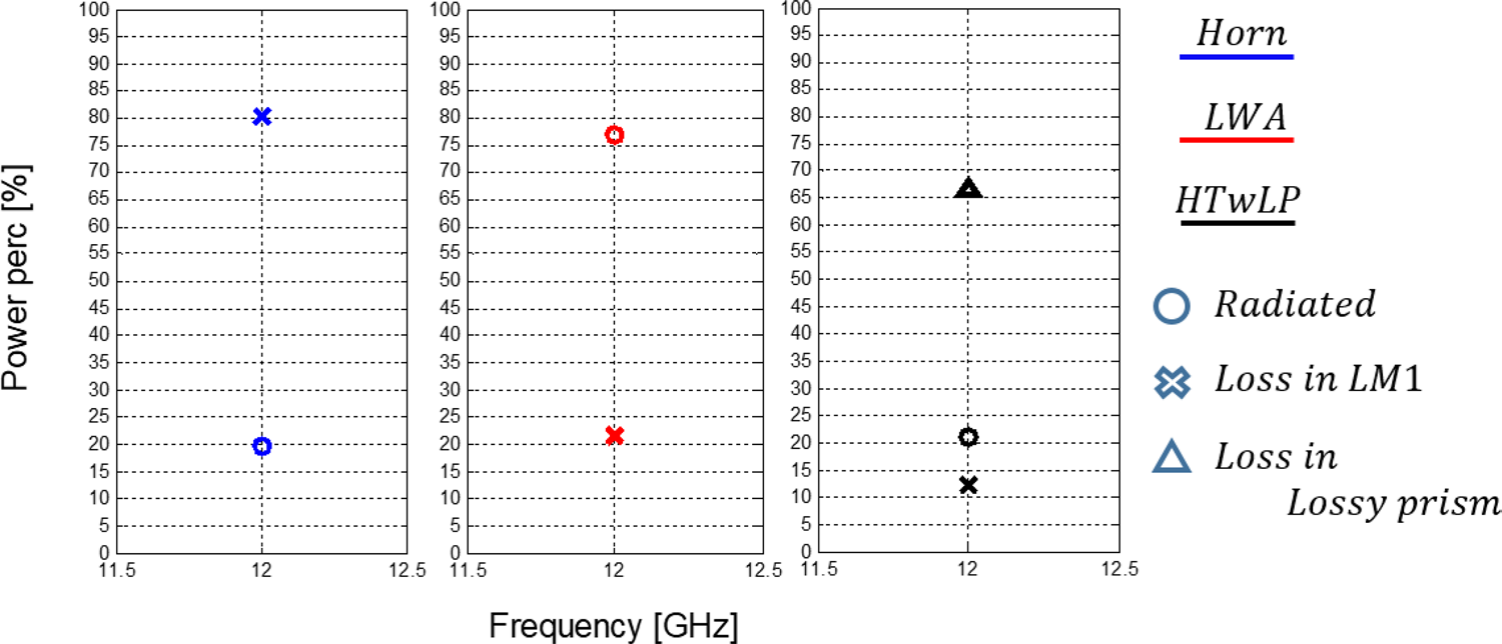
Percentage of power radiated and loss in the three cases.

If one would take the amount of power radiated at the aperture as a reference, he could see that more power is delivered within the LM1 by the horn antenna, as it is apparent in Fig. 11. Anyway, it is also clear that the power decreases much faster within the medium when the horn is considered. When practical applications suitable for the deep-penetration effects are considered, such as hyperthermia, this does not represent the best outcome, as the amount of maximum power delivered at the interface with LM1 has to be considered, instead, for obvious reasons (e.g., in order to fix a desired temperature or avoid to burn the surface). In those cases, a constant power/field at the interface must be imposed and it makes sense to consider the power/field within LM1 normalized by such a power/field. Doing so, we can see a clear advantage of the behavior exposed by the inhomogeneous radiators proposed.

## Methods

### Design of the horn antenna

Horns are medium-directive traveling-wave antennas^6,27^ widely used in many fields, and their low Voltage Standing Wave Ratio (VSWR), wideband and easy manufacturing make them very successful, especially at the microwave band.

A pyramidal horn antenna was designed and simulated, taking into account some aspects.

Firstly, we performed our simulations inside the radiating near-field (Fresnel) region and not inside the reactive near-field region: for a horn antenna, the reactive field is usually considered negligible already at a distance ${r}_{0}=\lambda$ from the aperture^7^, where the Fresnel region starts. The Fresnel region conventionally extends beyond the aperture up to a distance ${r}_{1}\le 2{D}^{2}/\lambda$, being D the maximum diameter of the antenna aperture. For larger distances, the antenna is usually considered as radiating in the far field. The upper boundary of the Fresnel region illustrated is based on a criterion which establishes the maximum phase error to ${\phi }_{e}=\pi /8$ rad. In our case it is $D=140$ mm at the operating frequency of $12$ GHz, therefore the near-field radiating region extends from $25$ mm to $1.6$ m from the antenna aperture. Even though the near field extends for approximately $64\lambda$ we performed the simulation putting the lossy medium at the very short distance of $r=3/2 \lambda =37.5$ mm.

This distance was chosen to comply with the LWA requirements illustrated in the next paragraph. The base of the lossy parallelepiped LM1 was chosen as large as the antenna aperture, in order to better approximate the infinite planar separation condition requested by the theoretical approach. However, the assumption of a lossy medium larger than the antenna aperture is relevant in many practical near-field applications, e.g. hyperthermia^3^ or Ground Penetrating Radar (GPR)^1^: in both such examples the lossy medium to be heated and explored, can be many times wider than the antenna used for the task.

### Design of the Menzel leaky-wave antenna

We modelled a planar, mono-dimensional, and non-periodic structure, derived from the Menzel^33^ antenna, which consists of a simple uniform microstrip line etched on a dielectric slab. This structure was selected because it is highly efficient, with only the EH1 leaky mode excited and the unwanted higher-order modes under cut-off condition.

The antenna was designed to radiate in a vacuum (${k}_{1}={k}_{0}$) at the frequency of $12$ GHz to allow deep penetration with an incident angle ${\xi }_{1}=\pi /4$ rad on the lossy medium, positioned parallel to the antenna aperture and previously named LM1. Given those requirements, ${\beta }_{1c}$ in Eq. (2) assumes the value:

15 $$\frac{{\beta }_{1c}}{{k}_{1}}=\frac{1}{\sqrt{2}}\sqrt{1+\sqrt{1+{\left[\frac{2\times 0.05}{2\pi \times 12\times {10}^{9}\times 8.85\times {10}^{-12}}\right]}^{2}}}\approx 1.0027879$$

and consequently, ${\alpha }_{1c}/{k}_{1}$ assumes the value:

16 $$\frac{{\alpha }_{1c}}{{k}_{1}}=\sqrt{{\left[\frac{{\beta }_{1c}}{{k}_{1}}\right]}^{2}-1}\approx \sqrt{{1.0027879}^{2}-1}\approx 0.0747234$$

It has to be noted that, when an LWA is considered, the value of ${\alpha }_{1}$ does not follow directly from the value of ${\beta }_{1}$ as it happens for the plane wave, this is because an LWA always produces a field which can be expressed as the sum of multiple plane waves: antenna parameters have to be tuned to match the specific ${\beta }_{1}$ and ${\alpha }_{1}$ values^8^. Uniform LWAs are usually designed to meet certain requirements, not in terms of amplitudes of phase and attenuation vectors as illustrated in Eqs. (15) and (16) but in terms of their longitudinal components, i.e. ${\beta }_{z}/{k}_{1}$ and ${\alpha }_{z}/{k}_{1}$, respectively, those values can easily be obtained once the radiation angle is known:

17 $$\frac{{\beta }_{z}}{{k}_{1}}\approx 1.0027879\frac{\sqrt{2}}{2}\approx 0.711$$

18 $$\frac{{\alpha }_{z}}{{k}_{1}}\approx 0.0747234\frac{\sqrt{2}}{2}\approx 0.0528$$

Here, we employed a well-established full-wave numerical approach first presented in^34^ and based on a mixed-potential integral equation in a unit-cell environment and solved by the Method of Moments (MoM)^35^ in the spatial domain through a triangular Delaunay mesh^22,^^36^: the proposed technique was designed for periodic structures but it applies also to uniform structures such as the one considered here. The implemented code assumes an infinite transverse section (i.e., along the *x*-axis) of the background grounded dielectric structure, therefore the provided results are affected by a certain degree of approximation when the structure is laterally truncated.

The obtained values for both ${\beta }_{z}/{k}_{1}$ and ${\alpha }_{z}/{k}_{1}$ of the EH1 leaky mode well approximated the ones requested by Eqs. (17) and (18), and specifically the following values were found:

19 $$\left\{\begin{array}{c}\frac{{\beta }_{z}}{{k}_{1}}=0.7088\\ \frac{{\alpha }_{z}}{{k}_{1}}=0.05234\end{array}\right.$$

The antenna was then designed in a simulator to radiate at least $94\mathrm{\%}$ of the power injected in input, i.e., $\frac{P\left(L\right)}{P\left(0\right)}=0.06$, and furtherly optimized. The remaining power was absorbed by a matched load placed at the termination. The relevant antenna length *L*, along the *z*-axis, obtained by employing the theoretical equations in^7^^,^^8^, is reported here:

20 $$L=-\frac{\lambda }{\frac{4\pi {\alpha }_{z}}{{k}_{0}}}ln\left[\frac{P\left(L\right)}{P\left(0\right)}\right]\approx 106~\mathrm{mm}$$

Further numerical optimizations led the longitudinal size on the $z\mathrm{ axis}$ to approximately $L=5\lambda =125$ mm: this value resulted about $\lambda /2$ longer than the approximated value in Eq. (20), thus leading to a ratio between radiated and injected power slightly higher than 94%. In our simulations, only the mode of interest was excited: a possible prototype would require the suppression of undesired modes. In the case of the considered LWA, the dominant EH_0_ bound mode can be suppressed feeding the structure with two symmetric stubs coming from below and soldered to the microstrip line. To excite the mode of interest, such two stubs must be excited with a phase difference of $\pi$ at the frequency of interest^7^. Otherwise one could think to use an asymmetric feed arrangement and cutting the currents on the microstrip with some transverse slits on the centerline^33^. Eventually, the suppression of the fundamental mode can be obtained through a vertical via connecting the central axis of the microstrip with the ground plane^7^: such a design is equivalent to considering just half of the Menzel antenna whose LWA radiation pattern is very well approximated by the one of the full antenna.

### Design of the horn TEM with lossy prism

The design of the horn TEM with lossy prism has to deal with several drawbacks. In^19^, an extensive study of the structure has been performed, and in^20^ we proposed a 2D model where the lossy prism began exactly where the horn TEM terminated. Here we inserted the horn TEM inside the prism as we noticed that in this way the surface waves at the interface air-lossy prism were limited.

We began the design by setting the ${\sigma }_{prism}$ using Eqs. (5) and (6), imposing α $={\alpha }_{1c}$, $\beta ={\beta }_{1c}$, $f=12$ GHz, $\xi =\frac{\pi }{4}$ and the refractive index ${n}_{prism}=1$. Here Eq. (6) written for ${\sigma }_{prism}$ is reported:

21 $${\sigma }_{prism}=\omega {\varepsilon }_{0} \frac{\mathrm{sin}2\xi }{2}\sqrt{{\left(2 {\left(\frac{{\alpha }_{1c}}{{k}_{0}}\right)}^{2}+1\right)}^{2}-1}={\sigma }_{LM1}=0.05\frac{\mathrm{S}}{\mathrm{m}}$$

The unitary values for relative permittivity ${\varepsilon }_{r}$ and permeability ${\mu }_{r}$ can, in practice, be accomplished by using foams carefully loaded with carbons or other dissipative materials. In this analysis, those values resulted useful to limit reflections at the interfaces between air and the lossy prism.

Therefore, the relative permittivity, relative permeability, and conductivity of the lossy prism are:

22 $$\begin{aligned} \varepsilon_{prism} & = 1 \\ \mu_{prism} & = 1 \\ \sigma_{prism} & = 0.05\, {\text{S/m}} \\ \end{aligned}$$

With this choice of ${\varepsilon }_{prism}$ and ${\mu }_{prism}$, the incidence angle of the horn TEM with the prism must be equal to ${\xi }_{inc}=\xi =\frac{\pi }{4}$ rad, and this is the angle at which the horn TEM is positioned with respect to the prism.

The horn TEM was designed considering some aspects: first, it has to be long enough to show a wave that can be locally approximated with a plane wave when impinging on the prism. Also, to prevent the scatter contribution of the prism wedges, the horn TEM was optimized to limit the portion of the field reaching them. The prism was designed much larger than the horn TEM to maintain the hypothesis of infinite prism to hold. The efficiency is reduced due to prism losses: this effect is more evident as the path that the wave has to pass through the prism is longer. Eventually, all the side lobes are due to the field scattered by the prism wedges.

## Supplementary Information

Supplementary Information.
