# Supplementary material for: Verification of the electromagnetic deep-penetration effect in the real world
Source: Sci Rep. 2021 Aug 5;11:15928. doi: 10.1038/s41598-021-95080-w (PMC8342490; doi:10.1038/s41598-021-95080-w)
Supplement: Supplementary file 1 — Supplementary Information. [file 41598_2021_95080_MOESM1_ESM.pdf]

Verification of the electromagnetic deep-penetration effect in the real world

Paolo Baccarelli[1,+], Alessandro Calcaterra[2,3,+], Fabrizio Frezza[2,+], Fabio Mangini[4,*], Nicholas Ricciardella[2], Patrizio Simeoni[2,5,+], Nicola Tedeschi[2,+]

1 Department of Engineering, Roma Tre University, 00146 Rome, Italy
2 Department of Information Engineering, Electronics and Telecommunications (DIET), Sapienza University of Rome, 00184 Rome, Italy
3 Elt Elettronica Group, 00131 Rome, Italy
4 Department of Information Engineering, University of Brescia, 25123 Brescia, Italy
5 National Transport Authority, Dún Scéine, Harcourt Lane, Dublin2, Dublin, Ireland
*fabio.mangini@unibs.it
+these authors contributed equally to this work

Supplementary information

1. ALGORITHM

With reference to Fig. 10 in the section "Results" of the paper.
To interpolate the calculated samples, we used the least-squares' algorithm [1].
For completeness, we evaluated the goodness of the calculated polynomial fits by reporting in Tab. 1 the root-mean-square (RMS) error and the mean-squared-error (MSE) that for the problem we are studying, coincide with the variance (σ^2). The entity of those values demonstrates that the behaviors of the samples are well described by polynomial interpolation.

	horn	Menzel antenna	HTwLP
RMSE	0.0077	0.0026	0.0060
σ^2	$5.98 \cdot 10^{-5}$	$0.67 \cdot 10^{-5}$	$3.56 \cdot 10^{-5}$

Tab. 1 - List of RMS error and variance for the polynomial fit performed in Fig. 10 of the paper.

In Tab. 1, RMS error and σ^2 were calculated with the following expressions:

$$\sigma^2 = \frac{\sum_{i=1}^{N}\left(E_{sample}(y_i) - E_{poly}(y_i)\right)^2}{N} \qquad RMS = \sqrt{\sigma^2} \tag{1}$$

2. ANTENNA DESIGN

We will report here information about the design and simulation of the antennas presented in the paper.

2.1 Horn antenna

The horn was designed assuring that the radiating near field occurred before the distance at which the prism was placed, i.e. at $y_{if} = 37.5$ mm [2,3]. Simulations were performed in time domain, including frequencies from the cut-off of the rectangular wave guide (occurring at 6.94 GHz for the chosen dimensions) to 13 GHz. The box was chosen so that to be distant at least $\frac{\lambda}{4}$ @ 12 GHz, that is the

frequency of interest. Eventually, a Perfect Magnetic Conductor (PMC) symmetry plane was placed at $x = 0$ mm, due to the symmetry of the guided and radiated field. Even if the symmetry is still guaranteed, the PMC plane was removed when simulations were performed in the presence of the lossy medium.
In Fig. 1 are shown the simulated models and the dimensions of the structure.
Fig. 2 represents the Voltage Standing-Wave Ration (VSWR) with and without the lossy medium. It can be noticed how reflections from the lossy medium are of little entity, not affecting the behavior of the horn antenna.

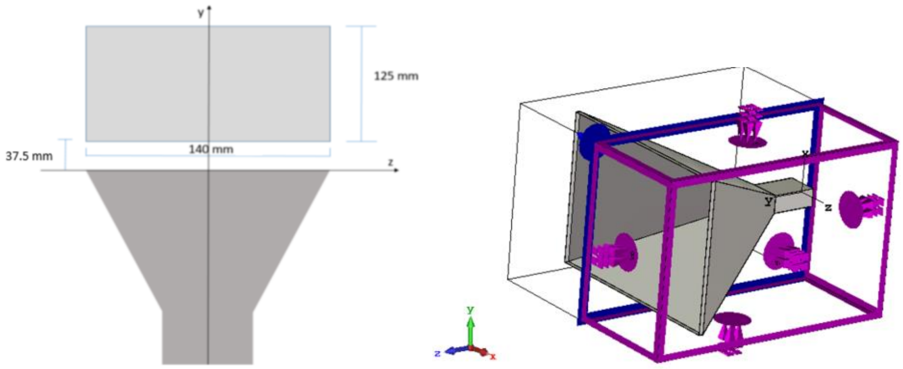

Fig. 1 - Horn antenna design on CST. Note the symmetric magnetic plane, in blue, passing through the centre of the antenna structure. Perfect Electric Conductor (PEC) was chosen as a medium for the antenna structure to allow faster simulations.

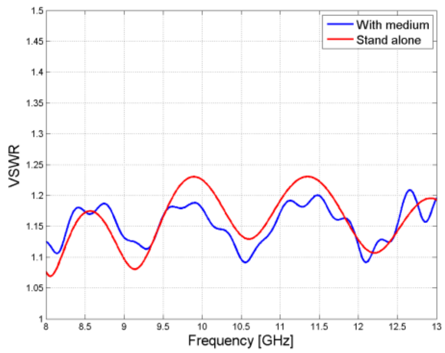

Fig. 2 – Voltage Standing-Wave Ratio (VSWR) with and without the medium posed in the near-field region. It can be seen how the presence of the prism does not create reflections that affect the antennas behaviour.

2.2 Menzel antenna

The Menzel antenna in Fig. 3 was designed to radiate an inhomogeneous wave at $\theta = \frac{\pi}{4}$ rad in vacuum, following design principles typical of leaky-wave antennas (LWAs) [3,4]. This design has been obtained calculating the dispersion diagrams shown in Fig. 4 [5] and then furtherly optimized by using a commercial full-wave simulator. The simulation of this structure is particularly demanding: the substrate is very thin with respect to the width of the strip, and this requires attention for the port dimension. The port dimension is important to be sure that the mode of interest is launched correctly in the leaky-wave antenna. Also, resonances in the structure can slow down convergence for some frequencies.
To perform this simulation, we imposed a mesh refinement at 12 GHz on S-parameters of at least 6 steps with two repetitions under a value of $\Delta = 0.01$. Also, the inhomogeneous port accuracy

enhancement has been activated. As can be seen in Fig. 3, a Perfect Electric Conductor (PEC) plane has been imposed on the longitudinal axis of the antenna. This allowed us to suppress the two modes below the one of interest. The antenna was designed to radiate at least 94% of the accepted power: in order to avoid the spared power being partially reflected and scattered at the end of the antenna, we placed an identical waveguide port in the terminal section to absorb it. In a real prototype, a matched load must be placed. For the simulation purposes, we could even have placed a lumped resistor accurately calculated to be matched to the impedance of the radiated mode or a lumped port with an internal impedance calculated in the same manner.
The three dimensional radiation pattern of the EH_1 leaky mode, according to the full-wave Finite Integration Technique (FIT) and Finite Element Method (FEM) simulations performed, is shown in Fig. 5 together with the radiation pattern at $\phi = \pi/2$ rad plane, and both are consistent with the results predicted on the basis of the Fortran numerical simulation in the design step.

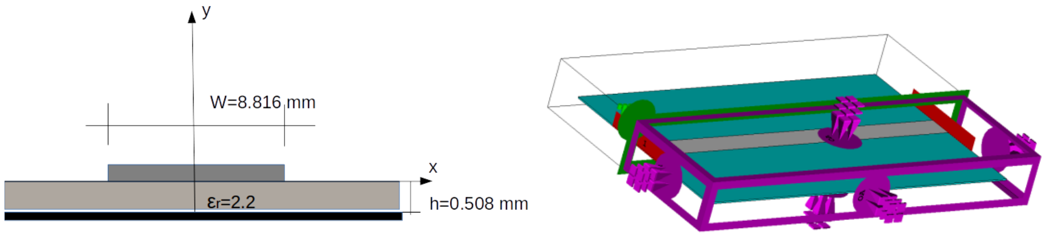

Fig. 3 – Microstrip LWA dimensions. Note that both the metal strip and the ground-plane are considered PEC and that the substrate losses are neglected, i.e. the substrate conductivity is assumed zero.

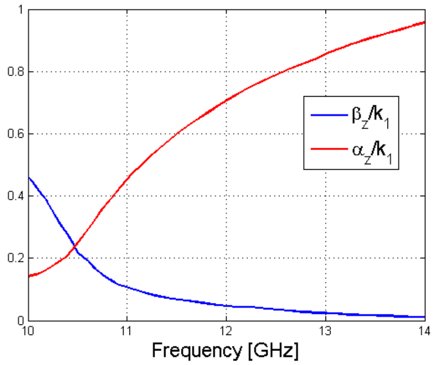

Fig 4 – Dispersion diagram computed in the LWA design: β_z/k_1 (in red) and α_z/k_1 (in blue) are shown.

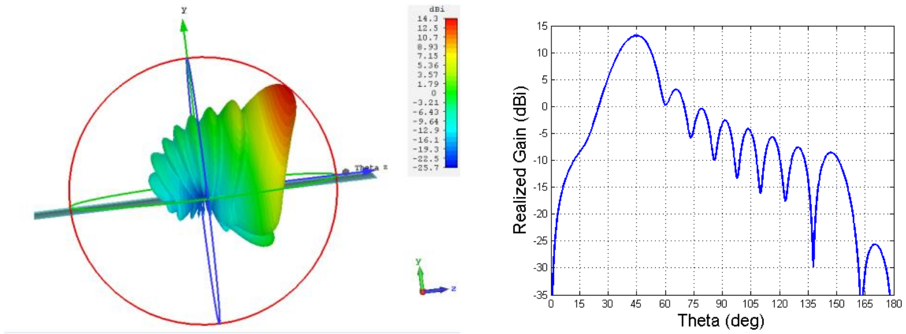

Fig 5 - Microstrip LWA radiation pattern at f = 12 GHz. *On the right, the pattern at the plane* $\phi = \frac{\pi}{2}$ rad*. Note the absence of back lobes due to the waveguide port placed as a load, the typical conical shape, and the presence of grating lobes.*

2.3 Horn TEM with Lossy prism (HTwLP)

The HTwLP has been designed with the principle of achieving an inhomogeneous wave by introducing losses with a lossy prism [6], as can be seen in Fig 6. The horn TEM instead of a simple horn antenna was chosen to assure a wave as much similar to a plane wave as possible. The antenna is visible in Fig. 7. To spare simulation-time, we placed a perfect magnetic plane along the $y = 0$ axis, due to the symmetry of the structure and of the mode of interest: the TEM mode. We could even have placed a perfect electric plane on the $x = 0$ plane, for the same reason. The realized gain is visible in Fig. 7. As every horn, the length of the antenna increases the uniformity of the phase on the aperture, resulting in a greater gain, due to the greater aperture efficiency. In this case, equivalently, the length has been chosen large enough to show a plane front in proximity of the interface with the lossy prism. Also, it can be noticed that the horn TEM is tilted to radiate at $\theta = \frac{\pi}{4}$ rad as the Menzel antenna and is inserted in the lossy prism (see Fig. 8). This choice limits the reflections.
It has been said in the paper that in order to maintain all the attenuation vector generated at the first interface illuminated by the field produced by the horn TEM, the lossy prism should form a $\frac{\pi}{2}$ rad angle at its upper edge. In this way, once the field is entered into the prism, it finds a second interface whose normal is orthogonal to its attenuation vector, and for the conservation of the parallel component of the fields $\bar{E}, \bar{H}$, all its module is conserved by passing in the third medium which is, again, air.
This $\pi/2$ -edge, by the way, is responsible for many scattering contributions that affect the total field. Placing the horn TEM antenna far from the wedge, would mean that the field has to pass through a longer path inside the lossy prism, being strongly attenuated. On the other hand, a longer horn TEM has a more focused pattern: this limits the interaction with the scattering wedge. All these factors were taken into account to achieve the final structure.
The prism was designed much larger than the horn TEM to allow the hypothesis of infinite prism to hold. It can be noticed in Fig. 8 how the lossy prism affects the horn TEM pattern, comparing the far-field diagram with the red curve of Fig. 7. The efficiency is reduced due to prism losses: this effect is more evident as the path that the wave has to pass through the prism is longer.
Eventually, all the side lobes are due to the field scattered by the prism wedges.

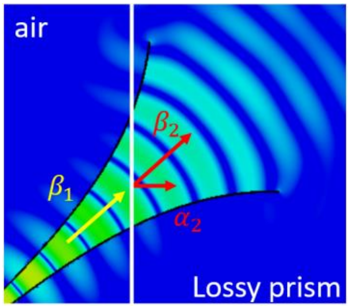

Fig. 6 - Interface between the horn TEM and the lossy prism. The horn TEM was designed long enough to accomplish the hypothesis of plane wave impinging on the prism.

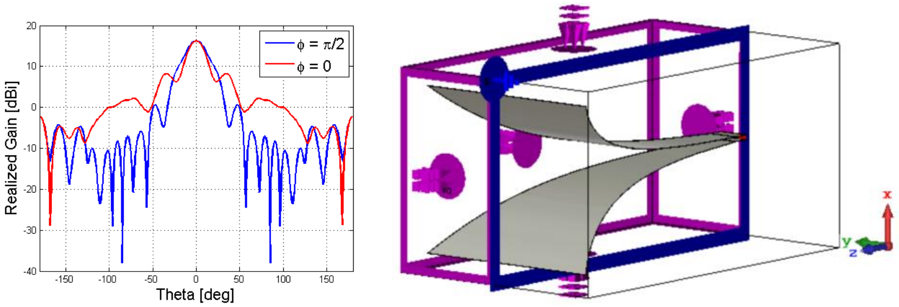

Fig. 7 – Design of the horn TEM antenna and Realized Gain on the E-plane ($\phi = 0$) and H-plane ($\phi = \frac{\pi}{2}$).

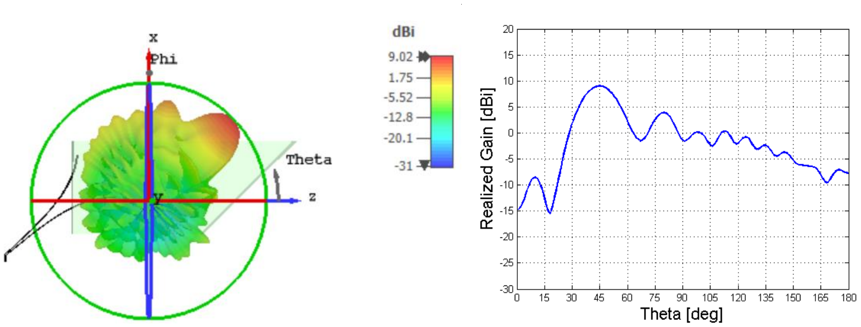

Fig. 8 - HTwLP pattern 3D and on the $\phi = 0$ plane. Note the presence of side lobes due to the scattering of the prism wedges.

REFERENCES

1. Charnes, A., Frome, E. L.; & Yu, P. L., The Equivalence of Generalized Least Squares and Maximum Likelihood Estimates in the Exponential Family. *Journal of the American Statistical Association* ***71***, 169–171 (1976).
2. Balanis, C. A. *Antenna Theory: Analysis and Design.* (John Wiley & Sons, New York, 2005).
3. Balanis, C. A. *Modern Antenna Handbook*. (John Wiley & Sons, New York, 2011).
4. Oliner, A. A., Jackson, D. R. & Volakis, J. *Antenna Engineering Handbook.* (McGraw Hill Professional, New York, 2015).
5. Baccarelli, P., Di Nallo, C., Paulotto, S. & Jackson, D. R. A full-wave numerical approach for modal analysis of 1D periodic microstrip structures. *IEEE Trans. Microw. Theory Tech.* **54**, (2006).
6. Frezza, F., Simeoni, P. & Tedeschi, N. Analytical investigation on a new approach for achieving deep penetration in a lossy medium: the lossy prism. *J. of Telecomm. and Inf. Tech.* (2017).
